# G_q_ rather than G_11_ preferentially mediates nociceptor sensitization

**DOI:** 10.1186/1744-8069-9-54

**Published:** 2013-10-25

**Authors:** Laura Nikita Wirotanseng, Rohini Kuner, Anke Tappe-Theodor

**Affiliations:** 1 Pharmacology Institut, University of Heidelberg, Im Neuenheimer Feld 366, Heidelberg, D-69120, Germany

## Abstract

**Background:**

The G_q/11_-protein signaling mechanism is essential throughout the nervous system, but little is known about the contribution of the individual G-protein GPCR signaling branches towards nociceptor activation and their specific role on nociceptor sensitization. We aimed to unravel the contribution of the G_q/11_-signaling pathway towards nociceptor activation via a variety of classical inflammatory mediators signalling via different G-protein GPCRs and investigated the specific contribution of the individual G_q_ and G_11_ G-Proteins in nociceptors.

**Findings:**

Using different transgenic mouse lines, lacking Gα_q_, Gα_11_ or both α-subunit of the G-proteins in primary nociceptive neurons, we analyzed the mechanical- and heat-sensitivity upon application of different GPCR-agonists that are known to play an important role under inflammatory conditions (e.g. ATP, Glutamate, Serotonin etc.). We found that the G_q/11_-GPCR signaling branch constitutes a primary role in the manifestation of mechanical allodynia and a minor role in the development of thermal hyperalgesia. Moreover, with respect to the mediators used here, the G_q_-protein is the principle G-protein among the G_q/11_-protein family in nociceptive neurons leading to nociceptor sensitization.

**Conclusions:**

Our results demonstrate that the G_q/11_ signaling branch plays a primary role in nociceptor sensitization upon stimulation with classical GPCR ligands, contributing primarily towards the development of mechanically allodynia. Moreover, the deletion of the individual G-proteins led to the finding that the G_q_-protein dominates the signalling machinery of the G_q/11_ family of G-proteins in nociceptive neurons.

## Findings

### Introduction

G-protein coupled receptors (GPCRs) represent the largest family of seven transmembrane receptors and downstream signaling constitutes one of the most important signaling pathways to regulate physiological processes. GPCR family members represent a major primary target for drug development [1,2] and their signaling is a predominant focus in the development of novel analgesic therapeutics [3].

Peripheral sensitization is accompanied by an inflammatory milieu, acting on receptors and channels on the peripheral nerve terminals (reviewed in [4]). Most of these sensitizers are known to bind to GPCRs of the G_q/11_ family, the G_i/o_, G_s_ and G_12/13_ family of heterotrimeric G-proteins.

We have recently elucidated the specific significance of the G_q/11_ pathway in modulating properties of nociceptors *in vivo* in the context of physiological pain and pathological states [5]. We found that G_q/11_ is involved in sensitization mechanisms in pathological states and tonically modulates basal nociception and acute pain [5].

There are four members of the G_q/11_-protein family, namely G_q_, G_11_, G_14_ and G_15/16,_ which activate Phospholipase C beta isoforms to regulate intracellular calcium. G_15/16_ overall show very low levels of expression whereas G_14_ has been shown to be expressed at high levels selectively in some tissues (e.g. kidney, lung and spleen; reviewed by [6]), and for the first time Han et al. showed that G_14_ is expressed in a subset of DRG neurons [7] but does not compensate for a loss of G_q/11_[5].

The aim of this study was to investigate the individual role of the G_q_ or G_11_ signaling branch towards acute nociceptive behavior induced by different GPCR ligands specifically activating G_q/11_-coupled GPCRs or GPCRs that are capable to couple different G-protein classes. This is the first study addressing the distinct roles of G_q_ and G_11_ towards nociceptor sensitization.

## Methods

All animal use procedures were in accordance with ethical guidelines imposed by the local governing body (Regierungspräsidium Karlsruhe, Germany). All behavioral measurements were done in awake, unrestrained, age-matched mice that were more than 3 months old, by individuals who were blinded to the genotype of the mice being analyzed. Genotypes were identified by genomic tail DNA PCR (as described earlier [5]). Animals were kept on a 12-hour light–dark cycle with constant room temperature and behavioral tests were performed in an appropriate quiet room between 11 am and 4 pm.

We used the following mice, which have been described in detail (except SNS-Gα_q_^-/-^ mice) before ([5,8]): Homozygous mice deficient for Gα_11_ (Gα_11_^-/-^) carrying the floxed allele of the mouse Gα_q_ (Gα_q_^fl/fl^) gene (SNS-Cre^-^;Gα_q_^fl/fl^;Gα_11_^-/-^; referred to as Gα_11_^-/-^ in this manuscript), sensory neuron-specific conditional double deficient mice for Gα_q_ and Gα_11_ (SNS-Cre^+^;Gα_q_^fl/fl^;Gα_11_^-/-^ mice; referred to as SNS-Gα_q/11_^-/-^ in this manuscript), sensory neuron-specific conditional single deficient mice for Gα_q_ (SNS-Cre^+^;Gα_q_^fl/fl^;Gα_11_^+/+^; referred to as SNS-Gα_q_^-/-^ in this manuscript) and mice carrying the floxed allele of Gα_q_ (Gα_q_^fl/fl^; referred to as control in this manuscript).

The following classical algogens and agonists were injected into the plantar surface of the hindpaw in a total volume of 20 μl: 5 μg Glutamate (27 nmol), 0.1 μg Bradykinin (94 nmol), 40 μg UTP (83 nmol), 5 μg CGRP (1.3 nmol), 1 μg mcPAF (1.85 nmol), 1 μg S1P (2.64 nmol), 60 μg ATP (0.1 μmol), 10 μg Serotonin (47 nmol), 50 ng PGE2 (142 nmol), 13U Trypsin, 1U Thrombin. Analysis of latency of paw withdrawal in response to heat was done, as previously described in detail ([5]; Plantar test apparatus, Ugo Basile Inc, Comerio, VA, Italy) and mechanical sensitivity was tested in the same cohort of animals via manual application of von Frey hairs to the plantar surface of the hind paw, as previously described in detail [5]. Two different substances were tested per mouse with 1–2 weeks of recovery period between the applications at different hindpaws. We used 6–8 mice per group, the exact numbers per group are given in Table 1 and the Figure legend.

**Table 1:** **Summary of behavioral results showing main impact of G**_**q**_**-mediating sensitization processes**

**Ligand**	**Receptor**	**G-protein subclass**	**Thermal hyperalgesia – paw withdrawal latency**				**Mechanical allodynia – paw withdrawal response frequency**			
			**(Mean % change over pre-injection)**				**(Mean Δ increase over pre-injection) 0.4 g von Frey Filament**			
			**Control**	**G**_**11**_^**-/-**^	**SNS-G**_**q/11**_^**-/-**^	**SNS-Gα**_**q**_^**-/-**^	**Control**	**G**_**11**_^**-/-**^	**SNS-G**_**q/11**_^**-/-**^	**SNS-Gα**_**q**_^**-/-**^
**Bradykinin**	B	G_q/11_	-51.5 ± 3.3 (n=8)	-37.1 ± 2.5 (n=7)	-7.4 ± 11.5* (n=7)	-8 ± 2.7* (n=7)	54.1 ± 5.5	27.8 ± 8*	14.4 ± 3.6*	6.7 ± 5.9*
**UTP**	P2Y	G_q/11_	-44.2 ± 10.4 (n=8)	-23.8 ± 7.2 (n=8)	-9.7 ± 7.5* (n=7)	-9.8 ± 5.1* (n=8)	32.8 ± 3.2	20 ± 8.9	4.8 ± 3.5*	6.7 ± 6.0*
**Trypsin**	PAR	G_q/11_	-41 ± 5.7 (n=8)	-38.3 ± 5 (n=8)	-17.7 ± 3.3* (n=8)	-14.3 ± 8.1* (n=6)	-3.3 ± 3.1	4.2 ± 1.8	0.8 ± 0.8	
**CGRP**	CGRP	G_q/11_, G_s_	-31,3 ± 6.9 (n=7)		-29.2 ± 2.3 (n=8)		21.1 ± 8.7		6.7 ± 2.7	
**ATP**	P2Y	G_q/11_, G_s_	-48.4 ± 4.6 (n=8)	-42.2 ± 6.1 (n=7)	-16.7 ± 5.5* (n=8)	-12.3 ± 4.3* (n=8)	27.5 ± 2	17.1 ± 6.5	4.8 ± 3.8*	5 ± 2.4*
**ET-1**	ET	G_q/11_, G_s_, G_12/13_	-39.5 ± 4 (n=8)	-42.3 ± 6.2 (n=7)	-21.7 ± 3.2* (n=8)	-25.5 ± 3.9* (n=7)	26.7 ± 8.5	26.7 ± 5.7	-0.8 ± 4.4*	4.8 ± 5*
**PGE**_**2**_	EP	G_q/11_, G_s_, G_i/o_	-26.8 ± 5.6 (n=8)	-22.2 ± 8.8 (n=8)	-6.6 ± 8 (n=8)	-8 ± 5.7 (n=7)	15.6 ± 5.8	7.5 ± 4.8	-0.8 ± 2.9*	4.8 ± 5*
**Serotonin**	5-HT	G_q/11_, G_s_, G_i/o_	-41.9 ± 3.9 (n=8)	-34.4 ± 4.8 (n=8)	-31.4 ± 3.1 (n=8)	-24 ± 3.2* (n=7)	29.2 ± 5.9	14.2 ± 5.1*	7.5 ± 2.3*	2.9 ± 2*
**Glutamate**	mGluR1,2	G_q/11_, G_i/o_	-45.1 ± 11.8 (n=9)	-8.2 ± 6.5 * (n=8)	-11.2 ± 4.5* (n=9)	-12.5 ± 6.7* (n=9)	19.3 ± 9.7	30.4 ± 8.7	3.3 ± 6.3	6 ± 4.7
**mcPAF**	PAF	G_q/11_, G_i/o_	-49 ± 1.8 (n=7)	-38.7 ± 4.8 (n=8)	-30.7 ± 5 (n=7)	-26.7 ± 8.3 (n=8)	30.5 ± 5.8	36.7 ± 7.4	-1 ± 3.1*	5.8 ± 5.6*
**Thrombin**	PAR	G_q/11_, G_i/o_, G_12/13_	-27.5 ± 6.2 (n=8)		-30.1 ± 5 (n=8)		20 ± 3.3		16.7 ± 8.2	
**S1P**	S1P	G_q/11_, G_i/o_, G_12/13_	-51.6 ± 5.7 (n=8)	-50.8 ± 5 (n=8)	-32.3 ± 4.5* (n=8)	-34.2 ± 7.3* (n=8)	23.3 ± 5	20 ± 7.1	4.2 ± 2.5*	0.8 ± 4.1*

Table displays the mean % change of paw withdrawal latency upon thermal stimulation within 90 min upon GPCR-ligand application to the hindpaw and the delta increase of paw withdrawal frequency upon mechanical stimulation with 0.4 g von Frey filament within 75 min upon GPCR-ligand application. *p<0.05 ANOVA, post-hoc Fisher’s test and boldface, indicates significant differences towards control mice. n = mice per group for thermal hyperalgesia and mechanical allodynia.

All data are presented as mean ± standard error of the mean (S.E.M.). For multiple comparisons, Analysis of Variance (ANOVA) for random measures was performed followed by post-hoc Bonferroni’s test.

## Results

**Figure 1:**
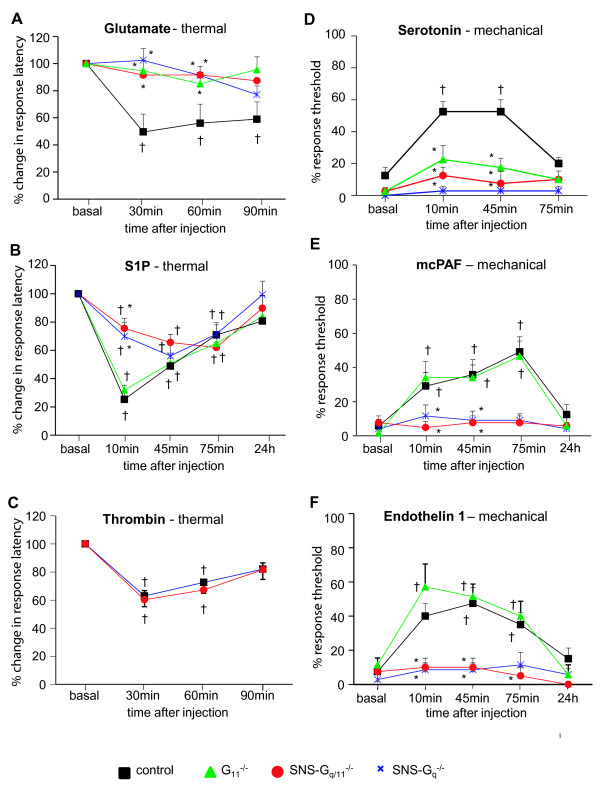
**Behavioral responses to intraplantar GPCR-ligands in control mice (black square symbols), G**_**11**_^**-/- **^**mice (green triangle symbols), SNS-G**_**q/11**_^**-/- **^**mice (red circular symbols) and SNS-G**_**q**_^**-/- **^**mice (blue cross symbols).** Magnitude and time course of hyperalgesia to plantar heat following unilateral intraplantar hindpaw injection of Glutamate (**A**; n= 9 for control, SNS-G_q_^-/-^ and SNS-G_q/11_^-/-^ mice and n= 8 for G_11_^-/-^ mice) S1P (**B**; n= 8 for all groups) and Thrombin (**C**; n= 8 for both groups) and of mechanical allodynia to mechanical von Frey filament stimulation following unilateral injection of Serotonin (**D**; n= 8 for control, G_11_^-/-^ and SNS-G_q/11_^-/-^ mice and n= 7 for SNS-G_q_^-/-^ mice), mcPAF (**E**; n= 8 for G_11_^-/-^ and SNS-G_q_^-/-^ mice and n= 7 for control and SNS-G_q/11_^-/-^ mice) Endothelin (**F**; n= 8 for control and SNS-G_q/11_^-/-^ mice and n= 7 for G_11_^-/-^ and SNS-G_q_^-/-^ mice). * *P*<0.05 as compared to the control group, ^†^ as compared to basal values within a group, ANOVA, post hoc Bonferroni’s test. All data points represent mean ± SEM.

The classical deletion of Gα_11_ led to a complete abrogation of Glutamate-induced thermal hyperalgesia (Figure 1A, Table 1) whereas mechanical hyperalgesia was entirely preserved (Table 1). We found a minor contribution of G_11_ towards Serotonin- induced mechanical hyperalgesia (Figure 1D, Table 1). Interestingly, thermal and mechanical hyperalgesia elicited by PGE_2_, Trypsin, Bradykinin, Endothelin1 (ET1), Sphingosin1 Phosphate (S1P), Platelet-activating factor (PAF), ATP, Thrombin and CGRP were completely preserved in G_11_-deficient mice (Figure 1B, 1C, 1E, 1F, Table 1).

We analyzed the algogen-induced behavior in G_q/11_ double deficient mice and found a complete loss of thermal hyperalgesia triggered by PGE_2_, Bradykinin, Glutamate, UTP and ATP, as well as mechanical hyperalgesia elicit by PGE_2_, Trypsin, Glutamate, UTP, Serotonin, ET1, S1P, PAF and ATP (Examples in Figure 1A, 1B, 1D-F, Table 1). There were minor changes with respect to thermal hyperalgesia upon ET1 and S1P application (Example in Figure 1B, Table 1), whereas thermal hyperalgesia towards Thrombin, CGRP and Serotonin and mechanical hyperalgesia towards Thrombin and CGRP was fully preserved in G_q/11_ double deficient mice (Example in Figure 1C, Table 1). Interestingly, the deletion of G_q/11_ in nociceptors had a stronger impact on mechanical allodynia than on thermal hyperalgesia.

Surprisingly, the single deletion of G_q_ caused the same behavioral phenotype as the double deletion of G_q_ and G_11,_ (examples in Figure 1, Table 1) indicating a predominant role for G_q_- over G_11_- proteins in nociceptive neurons.

## Discussion

We found that a particular G-protein pathway can contribute differentially to the action of diverse algogens and that a particular algogen can employ different G-protein pathways to elicit thermal hyperalgesia and mechanical allodynia. The G_q/11_ G-protein signaling pathway plays an important role for nociceptor sensitization and the transduction of GPCR signaling towards the development of mechanical allodynia and thermal hyperalgesia with respect to the mediators tested in this manuscript.

To our surprise G_q_ has a major impact over G_11_ mediated nociceptor sensitization. Although G_q_ and G_11_ are nearly ubiquitously expressed in overlapping patterns [9], including the dorsal root ganglia and spinal cord [7], it cannot be ruled out that specific, highly localized differences may exist between the expression pattern and subcellular distribution of G_q_ and G_11_ in central circuits mediating hyperalgesia. Previous studies showing no difference in receptor-coupling with respect to G_q_ or G_11_ are performed *in vitro*[10-13] and thereby might not reflect the *in vivo* situation. It is more likely that different expression levels as shown for different brain regions [14-17] or membrane compartmentalization might account for the observed phenotypes. With respect to the DRGs it seems that there is a signaling succession for members of the G_q/11_ family. G_15/16_ are not expressed, G_14_, G_11_ and G_q_ are expressed, while G_14_ has no specific role, G_11_ plays only a minor role for nociceptor sensitization and G_q_ is the most prominent G-proteins of this important signaling family. The classical deletion of G_q_ is known to be lethal [17], indicating essential requirement for this particular G-protein and no possible compensation of other G-proteins from different G-protein classes. Within the G_q/11_ G-protein class, a preferential signaling role of G_q_ over G_11_ signaling has been demonstrated in various systems [14-16,18-20] and the G_q_-protein mediated signaling pathway in DRGs seems to have the major role over all other possible G-protein pathways which are involved in signal transduction upon receptor activation after application of ligands. We used the Cre-lox P system for conditional deletion such that the gene deletion only commences prenatally, thereby excluding early developmental deficits in SNS-Gq^-/-^ mice, but we cannot rule out compensatory mechanisms of G_q_ in G_11_^-/-^ mice as it has been suggested earlier [21].

Moreover, we were surprised to see the predominant contribution of the G_q/11_ signaling pathway over G_s_ or G_i/o_ signaling with respect to those substances that are known to activate GPCRs which can bind different classes of G-protein, e.g. ATP, ET1, Glutamate, PAF, PGE_2_, Serotonin, S1P or Thrombin. Whereas the inhibitory G_i/o_ proteins contribute to anti-nociceptive signaling pathway, G_s_ and G_q/11_ protein signaling mediates pro-nociceptive signaling (reviewed in [3]). For example PGE_2_, a crucial mediator for inflammatory pain couples to G_q/11_-, G_i/o_- and G_s_-GPCRs but does not elicit thermal hyperalgesia or mechanical allodynia in mice lacking G_q,_ indicating a major contribution of the G_q_-GPCR signaling pathway. Similarly, ATP or Serotonin, which can bind G_q/11_- and G_s_-GPCRs, do not lead to mechanical allodynia in G_q_-deficient mice indicating a dominant role of G_q_ over the other G-proteins which are known to couple to the same receptors. On the contrary, thermal hyperalgesia and mechanical allodynia induced by CGRP (which can activate G_q/11_- and G_s_- coupled GPCRs) or Thrombin (which can bind G_q/11_- G_i/o_- and G_12/13_-GPCRs) are fully preserved, indicating that compensatory mechanisms via other G-proteins are functional.

Interestingly, with respect to thermal hyperalgesia only ATP-mediated heat hyperalgesia is abrogated in G_q_-deficient mice whereas Serotonin-induced heat hyperalgesia is preserved in these animals. This predominant role of the G_q_-protein in mediating mechanical allodynia over thermal hyperalgesia was also found for Endothelin and to some extend for S1P. It seems that the G_q/11_ signaling pathway contributes significantly to mechanical allodynia elicited via a broad range of inflammatory mediators herein tested and that GPCR agonist-induced heat hyperalgesia is mediated via distinct G-protein GPCRs or other receptors.

Our results constitute a valuable tool to work out *in vivo* conditions of established nociceptive sensitizers. Moreover, this tool can be used for studying the mechanisms of action of new mediators in pain sensitization.

## Competing interests

The authors declare that they have no competing interests.

## Authors’ contributions

LNW carried out behavioral experiments and analyzed results. RK provided general support and participated in the design of the study. ATT conceived and designed the study, carried out behavioral experiments, analyzed results and wrote the manuscript. All authors read and approved the final manuscript.
